# A novel de novo *HDAC8* missense mutation causing Cornelia de Lange syndrome

**DOI:** 10.1002/mgg3.1612

**Published:** 2021-08-03

**Authors:** Catia Mio, Nadia Passon, Federico Fogolari, Claudia Cesario, Antonio Novelli, Carla Pittini, Giuseppe Damante

**Affiliations:** ^1^ Department of Medicine (DAME) University of Udine Udine Italy; ^2^ Institute of Medical Genetics Academic Hospital of Udine Udine Italy; ^3^ Department of Mathematics Computer Sciences and Physics (DMIF) University of Udine Udine Italy; ^4^ Laboratory of Medical Genetics IRCCS Bambino Gesù Children Hospital Rome Italy; ^5^ Maternal and Child Health Department Academic Hospital of Udine Udine Italy

**Keywords:** Cornelia De Lange syndrome, *HDAC8*, molecular modeling, trio‐based exome sequencing

## Abstract

**Background:**

Cornelia de Lange syndrome (CdLS) is a rare and clinically variable syndrome characterized by growth impairment, multi‐organ anomalies, and a typical set of facial dysmorphisms. Here we describe a 2‐year‐old female child harboring a novel de novo missense variant in *HDAC8*, whose phenotypical score, according to the recent consensus on CdLS clinical diagnostic criteria, allowed the diagnosis of a non‐classic CdLS.

**Methods:**

Clinical exome sequencing was performed on the trio, identifying a de novo heterozygous variant in *HDAC8* (NM_018486; c. 356C>G p.Thr119Arg). Molecular modeling was performed to evaluate putative functional consequence of the HDAC8 protein.

**Results:**

The variant *HDAC8* c.356C>G is classified as pathogenic following the ACMG (American College of Medical Genetics and Genomics)/AMP (Association for Molecular Pathology) guidelines. By molecular modeling, we confirmed the deleterious effect of this variant, since the amino acid change compromises the conformational flexibility of the HDAC8 loop required for optimal catalytic function.

**Conclusion:**

We described a novel Thr119Arg mutation in HDAC8 in a patient displaying the major phenotypic traits of the CdLS. Our results suggest that a modest change outside an active site is capable of triggering global structural changes that propagate through the protein scaffold to the catalytic site, creating de facto haploinsufficiency.

## Graphical abstract

Here we describe a 2‐years‐old female child harboring a novel de novo missense variant in HDAC8, whose phenotypical score, according to the recent consensus on CDLS clinical diagnostic criteria, allowed the diagnosis of a non‐classic CdLS. The variant HDAC8 c.356C>G (p.Thr119Arg) is classified as pathogenic following the ACMG/AMP guidelines. By molecular modeling we assessed that the Thr119Arg mutation compromises the conformational flexibility of the HDAC8 loop required for optimal catalytic function.

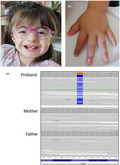

## 1 INTRODUCTION

The cohesion complex is a ring‐like chromatin‐associated multi‐subunit complex, highly conserved during evolution, mainly involved in sister chromatid cohesion but also in chromosome segregation, maintenance of genome stability, transcriptional regulation, chromatin structure, 3D genome organization, and DNA double‐strand break repair (Barbero, 2013; Watrin et al., 2016). Indeed, being an essential component of the family of modulators of chromatin structure, it is not surprising that defects in the cohesin complex or its regulators are associated to genetic instability syndromes, now called cohesinopathies (Sarogni et al., 2020).

Among these syndromes, the best defined is the Cornelia de Lange syndrome (CdLS), a multisystem malformation condition that due to its wide clinical variability includes five subtypes (OMIM #122470, # 300590, # 610759, # 614701, and # 300882), with either severe or milder phenotypes.

CdLS is a rare condition with a prevalence of 1/30’000 to 1/10’000 live births that is recognized primarily on the basis of characteristic facial dysmorphism and microcephaly, in association with prenatal and postnatal growth retardation, intellectual disability, and, in many cases, upper limb anomalies (Kline et al., 2018). CdLS display a huge clinical variability, with a wide spectrum of phenotypes ranging from mild to severe, with different degrees of facial and limb abnormalities even in the presence of variants affecting the same causative gene (Aoi et al., 2019; Avagliano et al., 2020).

Mutations in *NIPBL* (65%), *SMC1A* (5%), *SMC3* (<1%), *RAD21* (<1%), and *HDAC8* (4%) collectively account for about 75% of individuals with clinically diagnosed CdLS.

Heterozygous mutations in *NIPBL*, *RAD21*, and *SMC3* are usually transmitted in an autosomal dominant manner, while heterozygous or hemizygous mutations in *SMC1A* and *HDAC8* have a X‐linked related transmission (Infante et al., 2017). Most cases of CdLS are de novo (99%), with rare reports of inherited CdLS within families, but also cases of somatic mosaicism have been described (Barbero, 2013).

To date, 48 unique deleterious variants have been reported in *HDAC8*. It encodes for a class I metal‐dependent histone deacetylase that deacetylates SMC3 during S‐phase to facilitate renewal of the cohesin complex after its dissociation from chromatin (Mannini et al., 2013).

Here, we report a patient with typical CdLS harboring a previously unreported de novo *HDAC8* missense mutation, which was identified by trio‐based exome sequencing.

## 2 MATERIAL AND METHODS

### 2.1 Editorial policies and ethical considerations

Informed consent was obtained from all individuals included in the study.

### 2.2 Sample collection and whole‐exome sequencing (WES)

Genomic DNA was isolated from peripheral blood using the QIAsymphony DSP DNA Mini Kit (Qiagen, Hilden, Germany) following manufacturer's instructions. Library preparation and clinical exome capture were performed using the Twist Custom Panel kit (Twist Bioscience, San Francisco, CA, USA) and sequenced on the NovaSeq 6000 platform (Illumina). The BaseSpace pipeline (Illumina) and the TGex software (LifeMap Sciences) were used for variant calling and annotation. Reads were aligned to human genome build GRCh37/hg19. Based on the guidelines of the American College of Medical Genetics and Genomics and the Association for Molecular Pathology (ACMG/AMP), a minimum depth coverage of 20X was considered suitable for analysis. The mean coverage for the target region was 152X.

### 2.3 Homology modeling and molecular dynamic simulations

Molecular modeling and molecular dynamics simulations were performed as previously described (Mio et al., 2020). Briefly, the structure of the wild‐type HDAC8 was taken from the Protein Data Bank (www.rcsb.org). The entry with PDB id. 1t64 was chosen according to literature (Somoza et al., 2004). All ligands except Zn were removed and only chain A was considered. A model of the Thr119Arg mutant was obtained using the software Scwrl4 (Krivov et al., 2009) and keeping all residues different from the mutated one fixed in order not to modify the catalytic site. The resulting structure was soaked in a box of water and 0.150 M NaCl up to at least 14 A from any solute atom. The distance of the catalytic Zn from the coordinating atoms of the protein was restrained by a harmonic potential with a force constant equal to 400 kcal/mol A. All molecular simulations were performed using the program NAMD (Phillips et al., 2005). The structure was energy minimized in 5000 steps, heated to 310 K, and equilibrated at the same temperature for 1 ns. Then, 100 ns molecular dynamics was simulated by keeping pressure at 1 atm by a Langevin piston and keeping the temperature at 310 K by Langevin dynamics. The cutoff employed for non‐bonded interactions was 12 A, switching at 10 A and keeping track of all atoms within 14 A. The time step was 1 fs for bonded interactions and 2 fs for non‐bonded interactions. Hydrogen bond lengths were restrained by the algorithm settle. All details and relevant literature are provided in the user guide of the program NAMD (http://www.ks.uiuc.edu/Research/namd/cvs/ug.pdf). Exactly the same protocol was applied to the wild‐type and mutant protein.

## 3 RESULTS

The proposita is a 2‐year‐old Caucasian female referred to our Medical Genetics Institute for genetic counseling in the context of facial dysmorphisms, short hands and feet, mild limb hypertonia, cutaneous hemangiomas, and pulmonary valve dysplasia. She is the second‐born child of healthy non‐consanguineous parents. Family history was unremarkable. During prenatal screening, intrauterine growth restriction (IUGR), oligohydramnios, and decreased fetal movements (DFMs) were observed. C‐section was performed at 39 + 5 weeks of gestation. Birth weight was 2440 g (−2.5 SD), occipitofrontal circumference was 32 cm (−3 SD), and birth length was 45 cm (−3 SD). General physical examination showed full‐blown dysmorphic features: brachycephaly, low anterior hairline, arched eyebrows with synophrys, long eyelashes, broad nasal tip, smooth philtrum, and low‐set ears with overfolded helices of the left ear. She, also, presented a high‐arched palate, facial midline hemangioma, short neck, small hands and feet, pulmonary valve dysplasia, a low‐pitched growling cry, and developmental delay. Furthermore, infant audiological screening highlighted a congenital sensorineural hearing impairment. At 20 months, she was unable to sit without support, suggesting psychomotor development delay. Ophthalmologic evaluation disclosed visual impairment and myopia. Detailed clinical characteristics are enlisted in Table 1.

**TABLE 1:** Detailed phenotypical features of our proposita^a^

Clinical characteristics	HPO identifier	HDAC8+/−	Proposita
Growth			
Intrauterine growth retardation (IUGR)	HP:0001511	50–70%	Yes
weight below 5th percentile for age	HP:0004325	20–40%	Yes
height or length below 5th percentile for age	HP:0004322		Yes
Dysmorphic features			
Brachycephaly	HP:0001626	70%	Yes
Microcephaly	HP:0000252	29%	Yes
Low anterior hairline	HP:0000294	67%	Yes
Arched eyebrows	HP:0002553	88%	Yes
Synophrys	HP:0000664	≥90%	Yes
Long eyelashes	HP:0000527	45%	Yes
Telecanthus and/or hypertelorism	HP:0000506, HP:0000316	64%	Yes
Depressed nasal bridge	HP:0005280	45%	No
Anteverted nostrils	HP:0000463	76%	No
Long philtrum	HP:0000319	57%	No
Smooth philtrum	HP:0000319	30%	Yes
Thin upper vermilion lip	HP:0000219	20–50%	No
Downturned corners of the mouth	HP:0002714	57%	No
Highly arched palate	HP:0000218	20–50%	Yes
Small widely spaced teeth	HP:0000687	61%	Yes
Micrognathia/retrognathia	HP:0000347, HP:0000278	59%	No
Low‐set and/or malformed ears	HP:0000369, HP:0000377	20–50%	Yes
Short neck	HP:0000470	48%	Yes
Trunk and limb abnormalities			
Oligodactyly and adactyly (hands)	HP:0012165, HP:0009776	44%	No
Small hands and/or feet	HP:0200055, HP:0001773	≥90%	Yes
Proximally placed thumbs	HP:0009623	70–90%	No
5th finger clinodactyly	HP:0004209	50%	No
Short 5th finger	HP:0009237	84%	Yes
Neurosensory–Skin defects			
Ocular defects	HP:0000478	40–60%	Yes
Deafness or hearing loss	HP:0000365	59%	Yes
Hirsutism	HP:0001007	65%	No
Nevus flammeus	HP:0001052	58%	Yes
Development			
Developmental delay	HP:0001263	90%	Yes
Intellectual disability	HP:0001249	100%	Yes
Other			
Gastroesophageal reflux	HP:0002020	44%	NA
Feeding difficulties in infancy	HP:0011968	67%	Yes
Genitourinary anomalies	HP:0000119	36%	No
Cardiovascular anomalies	HP:0001626	40%	Yes
Behavior abnormalities	HP:0000708	20–50%	NA

^a^ Based on Gao et al. (2018), Kaiser et al. (2014), Kline et al. (2018).

**FIGURE 1:**
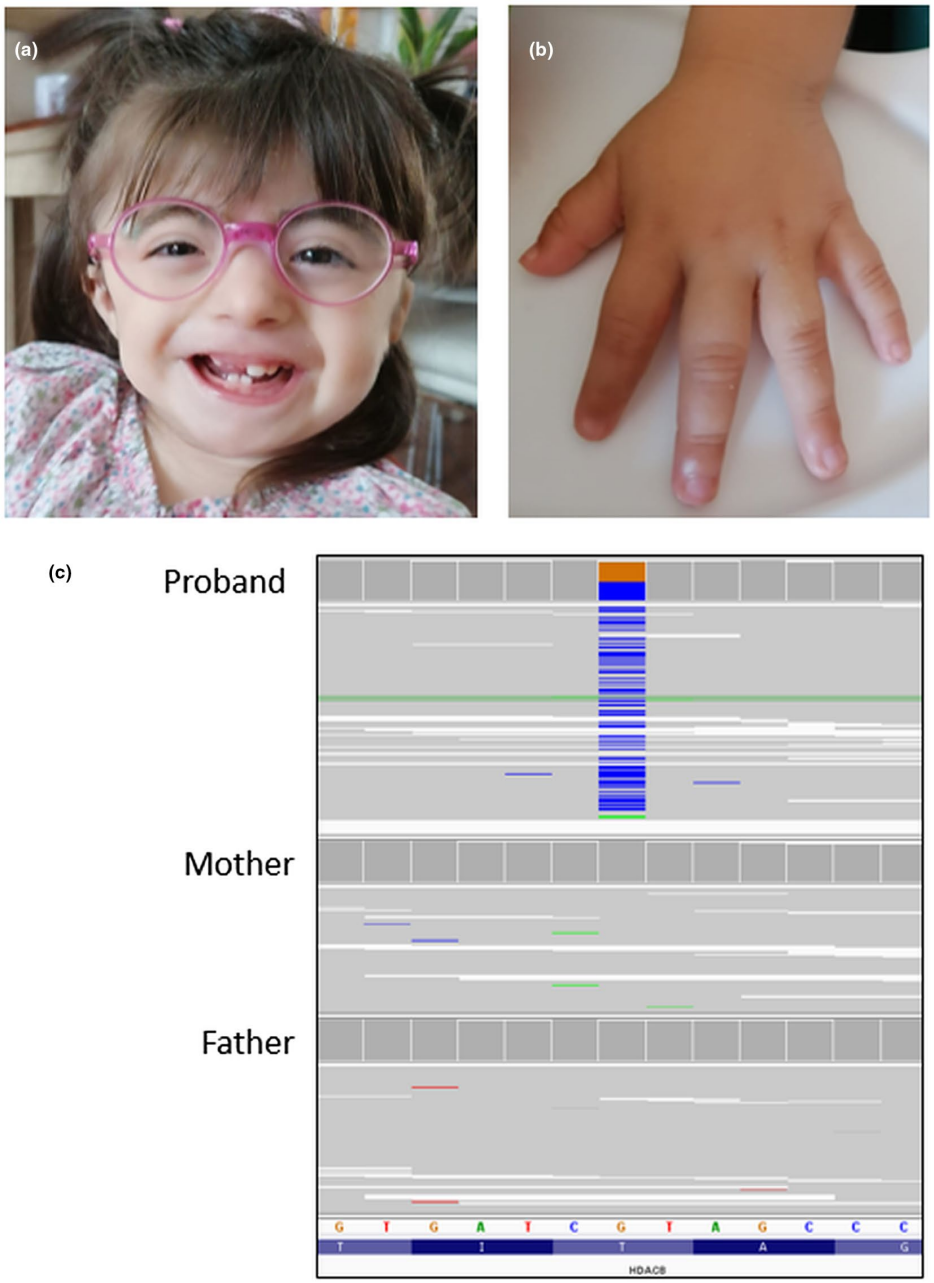
Phenotypic and genotypic characteristics of the proposita. (a) Facial dysmorphisms: low anterior hairline, arched eyebrows, hypertelorism, broad nasal tip, smooth philtrum, low‐set ears, widely spaced teeth, and thin upper lip; (b) Small hand with short fifth finger; c, NGS trio‐based analysis showing the de novo *HDAC8* (NM_018486) variant c.356C>G

Since the proposita's phenotype was suggestive of the CdLS (Figure 1a,b), clinical exome sequencing was performed on the trio, identifying a de novo heterozygous missense variant in the *HDAC8* gene (NM_018486) c. 356C>G (p.Thr119Arg) (Figure 1c). This variant is absent from the major population databases and results in a non‐conservative amino acid change located in the functional domain of the encoded protein. The substitution is never been reported and it is predicted as having a deleterious effect by multiple computational algorithms (15 of 17). Moreover, a C>T transition has been described on the same nucleotide by Yuan and colleagues (Yuan et al., 2015) and associated to CdLS. Based on the evidence outlined above and following the ACMG guidelines (Richards et al., 2015), the variant is classified as pathogenic (PS2, PM1, PM2, PP2, and PP3). We annotated the clinical classification for this alteration in the ClinVar database (RCV001268970).

**FIGURE 2:**
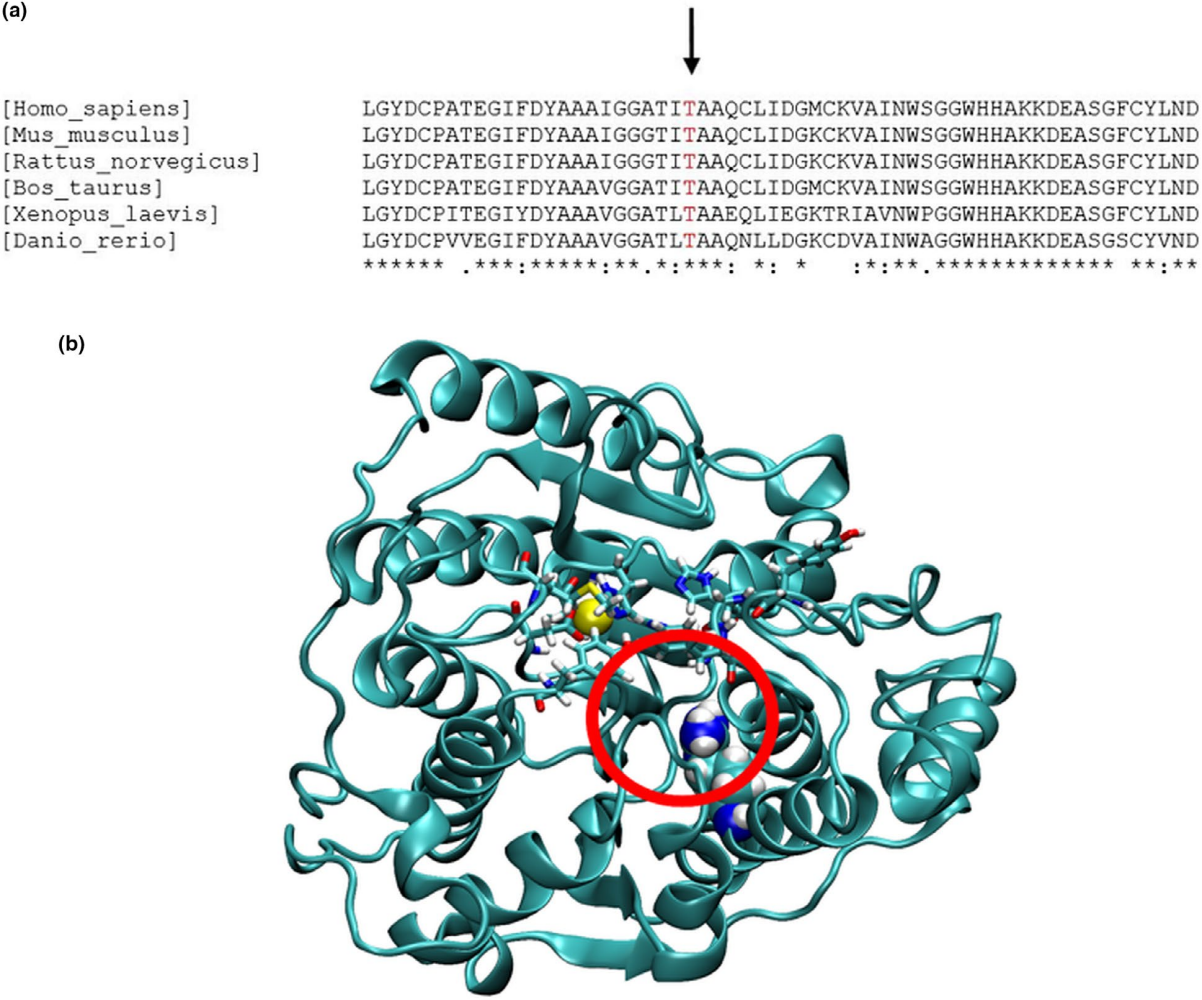
Sequence analysis and homology modeling of the HDAC8 mutant. (a) Partial protein alignment (aa 96–115) showing the conservation of the Thr119 residue (highlighted in red). (b) Residue 119 is shown as van der Waals spheres, the residues defining the binding and catalytic cavity are shown in licorice, and the zinc atom is shown as a yellow sphere. The position where Arg119 contacts the residue 140 adjacent to binding pocket residue is highlighted by a red circle

Relying on this, to assess the putative molecular consequences of the p.Thr119Arg substitution in HDAC8 amino acid sequence, molecular modeling was performed. Position 119 is located within one of the helices that participate in the three‐layer α‐β‐α domain, which spans the majority of the protein structure. The sequence, and in particular Thr119, is completely conserved from humans to frogs (Figure 2a).

The superposition of the secondary structure elements of the wild‐type and mutant protein after minimization resulted in an RMSD of 0.4 A, showing no substantial rearrangement in the three‐layer alpha‐beta‐alpha fold. Indeed, notwithstanding the largely different volumes of the two residues and the inner position of position 119, the mutation did not result in very severe steric hindrance. The snapshots taken from the molecular dynamics simulation runs were superimposed on the starting structure to check the structural variations along the trajectory. For both wild‐type and mutant, the average RMSD on the secondary structure elements was below 1.0 A, showing a rather stable arrangement on this timescale.

Although it is reasonable to expect that a buried charged residue like arginine would destabilize the folded form, in the simulations, the same residue establishes a salt bridge with Asp157. Twenty snapshots along the trajectory were superimposed onto each other using secondary structure elements and the RMSD of the Cα atoms of each residue was recorded and averaged. This procedure gave the RMSF at each residue. This was done for both proteins, and finally the snapshots from the wild‐type trajectory were superimposed in a similar fashion with those of the mutant trajectory.

The comparison of the within‐trajectory RMSFs with the between‐trajectory RMSFs highlighted the regions where the average RMSDs could not be justified by local fluctuations, that is, the two trajectories display fluctuations about different average conformations. The largest effect was observed at residues 138–140. This stretch of residues is adjacent to the residues 142–143 defining the catalytic pocket of the enzyme (Figure 2b).

## 4 DISCUSSION

The CdLS is a both genotypically and phenotypically heterogeneous disorder characterized by a core of dysmorphic features together with intellectual disability, growth delay, hirsutism, and limb defects. A recent consensus of a group of international experts has been made on clinical diagnostic criteria and molecular investigations (Kline et al., 2018). Our proposita's phenotype reached a score of 10, allowing the diagnosis of a non‐classic CdLS (Table 2).

**TABLE 2:** Clinical scoring for the diagnosis of Cornelia de Lange syndrome

Phenotypical feature	HPO identifier	Proposita
Cardinal^a^		
Synophrys and/or arched eyebrows	HP:0000664, HP:0000574	Yes
Short nose, concave nasal bridge, and/or upturned nasal tip	HP:0003196, HP:0011120, HP:0000463	No
Long and or smooth philtrum	HP:0000343, HP:0000319	Yes
Thin upper lip vermilion and/or downturned corners of mouth	HP:0000219, HP:0002714	No
Hand oligodactyly and/or adactyly	HP:0001180, HP:0009776	No
Congenital diaphragmatic hernia	HP:0000776	No
Suggestive^a^		
Prenatal growth restriction (<2 SD)	HP:0001511	Yes
Postnatal growth retardation (<2 SD)	HP:0008897	Yes
Global developmental delay and/or intellectual disability	HP:0001263, HP:0001249	Yes
Microcephaly	HP:0000252	Yes
Small hands and/or feet	HP:0200055, HP:0001773	Yes
Short fifth finger	HP:0009237	Yes
Hirsutism	HP:0001007	No
Clinical score^b^		10

^a^ Cardinal features are individually scored with 2 points; suggestive features are individually scored with 1 point.

^b^ ≥11 points (at least three cardinal): classic CdLS; 9 or 10 points (at least two cardinal): non‐classic CdLS; 4–8 points (at least one cardinal): molecular testing required; <4 points: insufficient evidence for molecular testing. Following the recommendations in Kline et al. (2018).

To uncover the molecular defect underlying the patient's phenotype, a trio‐based NGS approach was performed, highlighting a de novo heterozygous missense variant in *HDAC8*. *HDAC8* (OMIM #300269) is located on chromosome Xq13.1 and it encodes for a class I histone deacetylase that deacetylates SMC3 during S‐phase to establish the proper dissolution of pro‐cohesive elements and, consequently, the establishment of a new functional complex (Mannini et al., 2013). To date, more than 60 patients harboring a unique *HDAC8* mutation and showing cardinal features of the CdLS have been reported (Gao et al., 2018; Jezela‐Stanek et al., 2019).

Patients with *HDAC8* variants often not only show a CdLS phenotype, but also distinct features such as hypertelorism, a broad or bulbous nasal tip, hooding of eyelids, late closure of the anterior fontanelle, teeth anomalies, and mosaic patches of skin pigmentation. Moreover, it is already established that the severity of clinical phenotypes caused by *HDAC8* mutations is heavily influenced by random X‐inactivation in females (Kaiser et al., 2014).

Indeed, males are mostly severely affected, while females, who represent more than 70% of the patients (Parenti et al., 2016), display diversified phenotypes whose variability would be related to the extent of the tissue‐specific skewed lyonization (Parenti et al., 2016). Relying on this, it was not surprising that our proposita displayed a non‐classical CdLS.

To shed light on the molecular consequences underlying the identified mutation, homology modeling and molecular dynamics were performed. The mutation Thr119Arg is accommodated with small structural rearrangements, something which was not anticipated based on the largely different volume of the two amino acids and the internal position of the residue. It is likely that the mutation destabilizes the folded form due to a large entropic contribution associated with restrictions in conformational mobility. Arg119 is able to establish a favorable ionic interaction with Asp157 likely to balance desolvation free energy. The contact between Arg119 and the region 138–140 leads to structural sizeable differences. The region is located in one of the loops defining the substrate binding pocket. This suggests that the mutation can alter the binding of the substrate and, consequently, the catalytic efficiency of the protein. The effects observed are similar to those described by Decroos and colleagues regarding the p.Gly117Glu replacement, which compromises the conformational flexibility of the HDAC8 loop required for optimal catalytic function by triggering structural changes propagating through the protein scaffolding to the aforementioned loop. This substitution, in fact, resulted in a 5% residual enzyme activity (Decroos et al., 2015).

In summary, we described a novel Thr119Arg mutation in HDAC8 in a patient displaying the chief phenotypical characteristics of the CdLS, that is, brachycephaly, low anterior hairline, arched eyebrows with synophrys, long eyelashes, broad nasal tip, smooth philtrum, low‐set ears, short neck, small hands and feet, and a low‐pitched growling cry and developmental delay. Our computational data suggest that the amino acid change triggers a structural change that alters the substrate binging capability of the protein.

## CONFLICT OF INTEREST

The authors have no conflict of interest to declare.

## Data Availability

Data sharing is not applicable to this article as no new data were created or analyzed in this study.
